# Carrier Transfer and Capture Kinetics of the TiO_2_/Ag_2_V_4_O_11_ Photocatalyst

**DOI:** 10.3390/nano10050828

**Published:** 2020-04-27

**Authors:** Yun Zhou, Qiujie Ding, Yuan Wang, Xiaoping OuYang, Lixin Liu, Junyu Li, Bing Wang

**Affiliations:** 1 School of Materials Science and Engineering, Xiangtan University, Xiangtan 411105, China; zhouyun720@126.com (Y.Z.); xtdxdqj@sina.com (Q.D.); li_junyu@nuaa.edu.cn (J.L.); wangbing2015@aliyun.com (B.W.); 2 Institute of Fluid Physics, China Academy of Engineering Physics, P.O. Box 919-111, Mianyang 621900, China; liulix00@gmail.com

**Keywords:** TiO_2_/Ag_2_V_4_O_11_, nanoheterojunctions, photocatalyst, interface effect, carrier capture, recombination kinetics

## Abstract

In this paper, TiO_2_/Ag_2_V_4_O_11_ nanoheterojunctions have been synthesized by hydrothermal methods, which show enhanced photocatalytic activity compared to TiO_2_ under visible light. Moreover, the TiO_2_/Ag_2_V_4_O_11_ nanoheterojunction with set molar ratio of 2:1, referred to as TA2, show the highest visible light photocatalytic activity, which could decompose about 100% RhB molecules within 80 min of irradiation with visible light. Specially, the time-resolved photoluminescence spectrum of TA2 demonstrates that the free exciton recombination occurs in approximately 1.7 ns, and the time scale for Shockley–Read–Hall recombination of photogenerated electrons and holes is prolonged to 6.84 ns. The prolonged timescale of TA2 compared to TiO_2_ and Ag_2_V_4_O_11_ can be attributed to the carrier separation between nanojunctions and the carrier capture by interfacial defects. Furthermore, the enhanced photocatalytic activity of TiO_2_/Ag_2_V_4_O_11_ nanoheterojunctions also benefits from the synergistic effect of the broadened absorption region, higher photocarrier generation, longer carrier lifetime, and quicker collection dynamics.

## 1. Introduction

As is known, photocatalysis technology is considered as the most promising way to solve energy shortage and environmental pollution issues [1,2]. TiO_2_ has been widely researched as a potential photocatalyst for its high photocatalytic activity, non-toxic, inert chemical properties, and low cost. However, further application of TiO_2_ is unfortunately limited by the fast recombination of photogenerated carriers. Therefore, promoting the separation of photogenerated electron–hole pairs and increasing the utilization rate of photogenerated carriers are persistent goals [3,4]. In particular, appropriate heterojunctions are important structures for effectively promoting the separation of photogenerated electron–hole pairs [5,6,7]. When forming a heterojunction, two coupled semiconductors with a different Femi energy band (FB) could align favorably, resulting in an energy ladder between the conduction band (CB) and the valence band (VB) of the two semiconductors. As a result, the photogenerated electrons and holes can transfer in separate directions across the heterojunction boundary owing to the energy ladder between the band structure. Many successful nanoheterojunctions with excellent photocatalytic performance compared to their individual materials have been reported, such as g-C_3_N_4_/TiO_2_, TiO_2_/MoS_2_, CdS/WO_3_, and NiO/TiO_2_ [8,9,10,11]. It is confirmed that constructing a nanoheterostucture can effectively broaden the visible light absorption ability, inhibit the photogenerated carrier recombination, and ultimately, enhance the photocatalytic activity. In particular, Ag_2_V_4_O_11_ is a typical semiconductor with a narrow bandgap of approximately 2.0 eV. As reported, Ag_2_V_4_O_11_ shows outstanding electronic performance and is widely researched in the battery, gas sensor, and photocatalyst [12,13,14]. Thus, Ag_2_V_4_O_11_ is an ideal choice for constructing an effective nanoheterostructure with TiO_2_ to improve the photocatalytic activity under visible light. Herein, TiO_2_/Ag_2_V_4_O_11_ nanoheterojunctions are constructed in the paper to promote the separation of photogenerated carriers and reduce the carrier recombination. Additionally, in competition to the transfer and separation, photogenerated electrons and holes may quench by a direct band-to-band transition or Shockley–Read–Hall (SRH) recombination in the heterostructure. Furthermore, the transfer and separation kinetic of photogenerated carriers would be evidently influenced by the morphology and structure of the nanoheterojunction. Except for carrier recombination, there are also many other complex processes that can occur during the carrier transfer and diffusion process, such as charge localization, charge capture and non-radiation relaxation [15,16,17]. Therefore, tracking the transportation and quenching kinetics of photogenerated carriers in heterojunctions is a critical are of research that must be investigated in depth.

Time-resolved photoluminescence (TRPL) technology provides an effective method for measuring the carrier’s lifetime and reflecting the kinetics of photogenerated carrier [18]. For instance, Yamada et al. found that the diffusion process plays an important role in the kinetics of photocarriers in CH_3_NH_3_ PbBr_3_ perovskite by TRPL spectra [19]. Wang et al. revealed that anatase/rutile TiO_2_ phase junction can promote separation of photoinduced carriers on microsecond-time scales, but does not affect the charge recombination on a millisecond-time scale [20]. Li reported that the PL lifetime of the In_2_S_3_/TiO_2_ heterostructures is prolonged (compared 4.64 ns) compared to that of In_2_S_3_ (3.15 ns), indicating the effective photogenerated electron transfer from the CB of the In_2_S_3_ to the CB of the TiO_2_ [21]. Komarala et al. pointed that the radiative recombination of CuInS_2_/ZnS is related to the defects and surface-related trap states [22]. Herein, the photogenerated carriers’ kinetics of the of TiO_2_/Ag_2_V_4_O_11_ can be effectively obtained upon analysis of the TRPL spectra. The research of photogenerated carriers’ kinetics is of great significance to the cognition of the photocatalytic mechanism.

In this paper, TiO_2_/Ag_2_V_4_O_11_ nanoheterojunctions with enhanced visible light photocatalytic activity were successfully fabricated. Moreover, the TRPL spectra of TiO_2_/Ag_2_V_4_O_11_ nanoheterojunctions demonstrate that TiO_2_/Ag_2_V_4_O_11_ nanoheterojunctions show a longer carrier lifetime than TiO_2_ and Ag_2_V_4_O_11_, which can be ascribed to the faster carrier transfer owing to the nanoheterojunction’s effect and increased carrier capture because of the additional interfacial defect. The research reveals the photogenerated carriers’ kinetics in TiO_2_/Ag_2_V_4_O_11_ nanoheterojunctions and investigates the influence of defects, which are beneficial to deeply understand the enhancement mechanism of nanoheterojunctions and guide further design of nanoheterojunctions with higher photocatalytic activity.

## 2. Material and Methods 

### 2.1. Materials

TiO_2_ nanoparticles were purchased from XFNANO company (Nanjing, China). AgNO_3_ and NH_4_VO_3_ were purchased from Alfa Aesar (Shanghai, China) and were analytically pure. Deionized water was purchased from Aladdin (Shanghai, China). All chemicals were used without further purification. 

### 2.2. Fabrication of TiO_2_/Ag_2_V_4_O_11_

**Figure 1:**
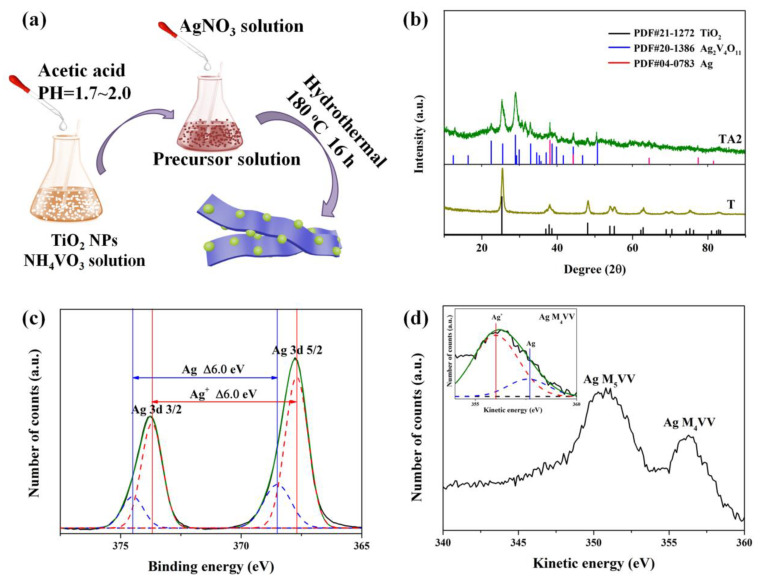
(**a**) Schematic of TiO_2_/Ag_2_V_4_O_11_ nanoheterojunction’s synthesis process, (**b**) XRD patterns of TiO_2_ (T) and TiO_2_/Ag_2_V_4_O_11_ nanoheterojunction with set molar ratio of 2:1 (TA2) samples, XPS high-resolution spectra of (**c**) Ag 3d and (**d**) Ag MVV of the TA2 sample.

Take the TiO_2_/Ag_2_V_4_O_11_ nanoheterojunctions with a molar ratio of 1:1 as an example to describe sample preparation. As shown in Figure 1a, 1 mmol NH_4_VO_3_ and 0.25 mmol TiO_2_ nanoparticles were dissolved in 20 mL deionized (DI) water at 40 °C and regulated the PH to 1.7–2.0 using acetic acid. Then, the solution composed of 20 mL DI water and 1 mmol AgNO_3_ was added dropwise into the above solution to form the precursor solution. Finally, the precursor solution was transferred into a 50 mL Teflon-lined autoclave and heated at 180 °C for 16 h, the obtained production was washed by DI water and dried at 70 °C to obtain the TiO_2_/Ag_2_V_4_O_11_ nanoheterojunctions. The added amount of TiO_2_ nanoparticles is 0.25 mol, 0.5 mol, 1 mol, and 2 mol for synthesis of TiO_2_/Ag_2_V_4_O_11_ with molar ratio of 1:1, 2:1, 4:1, and 8:1, respectively. In this paper, TiO_2_ nanoparticles, Ag_2_V_4_O_11_ nannobelts, and TiO_2_/Ag_2_V_4_O_11_ nanoheterojunctions with a set molar ratio of 1:1, 2:1, 4:1, and 8:1 were recorded as T, A, TA1, TA2, TA4, TA8, respectively.

### 2.3. Characterizations

The crystal structure and composition of the sample were characterized by X-ray diffraction (XRD, X’pert Pro, Cu Kα, λ = 1.5406, Panalytical Inc., Almelo, Netherlands), X-ray photoelectron spectroscopy (XPS, ESCALAB 250XI, Al K Alpha, E = 1486.6 eV, Thermo Scientific Ind., Waltham, MA, USA), and Raman spectra (InVia, Renishaw Inc., Gloucestershire, UK). The microscopic morphology of the sample was observed through field-emission scanning electron microscopy (FESEM, Hitachi S-4800, Carl zesis NTS GmbH Inc., Baden Wuerttemberg, Germany). Transmission electron microscopy (TEM, Libra 200FE, Carl Zeiss irts Inc. Baden Wuerttemberg, Germany) and high-resolution transmission electron microscopy (HRTEM) were used to further confirm the morphology and heterojunction interface. The photocatalytic activity of samples was characterized by the degradation of RhB molecules under visible light (Xe lamp: λ > 420 nm). Specifically, 50 mg catalysts were used for decomposing 30 mL RhB (10 mg L^−1^). During the photocatalytic reaction process, 3 mL aliquots were sampled at the time intervals of 20 min and filtrated to remove the catalysts. The variation of RhB concentrations were analyzed by recording the variations of the absorption band maximum (554 nm) of filtrate using a TU-1901 spectrophotometer. The sample is stirred to form paste with ethanol and coated onto the glass, then dried to obtain the working electrode. Photocurrent measurements were carried out under visible light using a three-electrode system on an electrochemical workstation (CHI-660B, CH Instruments Ins. Shanghai, China), where the sample, saturated calomel electrode (SCE), and platinum wire were set as the working electrode, reference electrode, and the counter electrode, respectively. The photo-response was measured with 0.1M Na_2_SO_4_ aqueous (PH = 7.0) as the electrolyte solutions at 0.5 V vs. SCE electrode under on–off cycles of simulated visible light. The Mott–Schottky plot was obtained by the three-electrode system with an electronic chemistry workstation (RST5200, ShiRuiSi Ins., Zhengzhou, China), where the sample disk formed under 4.5 GPa pressure, Ag/AgCl electrode, and platinum wire were set as the working electrode, reference electrode, and the counter electrode, respectively. The absorption spectra were recorded using a UV-Vis-NIR spectrophotometer (Solidspec-3700, Shimadzu Ins., Tokyo, Japan). The Fermi energy level was obtained by measuring the energy difference between the Rh probe and sample surface at room temperature through Kelvin probe force microscopy (KPFM, SII E-Sweep, Hitachi Ltd., Tokyo, Japan). Electron paramagnetic resonance spectroscopy (EPR) of samples was recorded at room temperature with a microwave frequency of 9.163 GHz (EMX-10/12, Bruker Co. Karlsruhe, Germany). The photoluminescence (PL) was tested under 532 nm light excitation at room temperature (F4600, Hitachi Ltd., Tokyo, Japan). Time-resolved PL spectra were obtained at 600 nm with an excitation wavelength at 532 nm at room temperature (TRPL, FLS980, Edinburgh Ins., England).

## 3. Results and Discussion

### 3.1. Characterization and Photocatalytic Performances

As shown in Figure 1b, we can see that the diffraction peaks of TiO_2_ (T) located at 25.3, 37.8, 48.0, 53.9, 55.1, and 62.7 correspond to the anatase TiO_2_ (PDF#21-1272). As for TA2, except for peaks of TiO_2_, the diffraction peaks located at 25.6, 29.0, 33.0, 38.7, and 50.7 are indexed to the monoclinic Ag_2_V_4_O_11_ (PDF#20-1386) diffraction pattern. Furthermore, the characterized peaks located at 38.1, 44.3, and 64.4 are attributed to the metal Ag (PDF#04-0783). The high-resolution XPS spectrum of Ag 3d is displayed in Figure 1c, which demonstrates that the silver species in the TA2 include Ag^+^ and metallic Ag [23]. Ag MVV spectrum of TA2 is employed to further investigate the Ag oxidation state, as displayed in Figure 1d. Moreover, the Auger parameter is crucial for estimating the Ag oxidation state. As is reported, the Auger parameter (α) is defined as the sum of the kinetic energy of the Auger electron (Ag M_4_VV) and the binding energy of the core level (Ag 3d5/2), and the values are about 726.1 and 724.4 eV for Ag and Ag^+^, respectively [24,25]. As for our TA2 nanoheterojunctions, the Auger parameter (α) is calculated to be 724.1eV, which is between the range of 726.1~724.4eV and approaches 724.4 eV, indicating the silver species in the TA2 are mainly Ag^+^ and a small amount of metallic Ag. According to the fitted peaks of Ag 3d5/2 and the Auger parameter, the Ag M_4_VV peak of TA2 is fitted as Ag and Ag^+^ peaks, as presented in the inset figure of Figure 1d. The XPS results reveal the co-existence of the Ag and Ag^+^ in the sample; these are entirely consistent with the XRD results.

**Figure 2:**
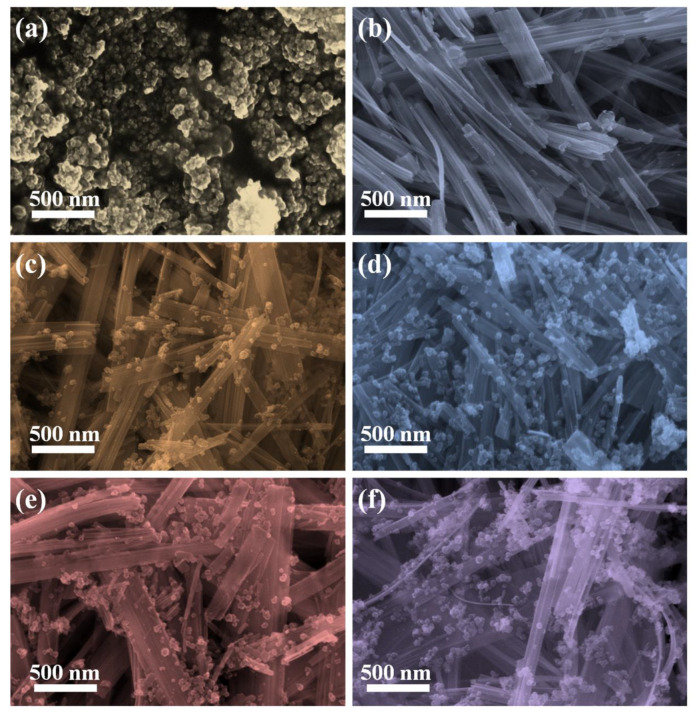
(**a**–**f**) SEM images of TiO_2_, Ag_2_V_4_O_11_ and TiO_2_/Ag_2_V_4_O_11_ nannoheterojunctions with set molar ratio of 1:1, 2:1,4:1 and 8:1 samples (recorded as T, A, TA1, TA2, TA4, and TA8), respectively.

Additionally, the morphology of samples is observed in the SEM images displayed in Figure 2. Pure TiO_2_ particles with a diameter of approximately 25 nm are agglomerated together, such agglomeration is not beneficial for the photocatalytic performance (Figure 2a). Figure 2b shows the morphology of A, which presents a one-dimensional nanobelt morphology, and the length of the nanobelt is several micrometers and the width ranges from 50 nm to 200 nm. The surface of A is relatively smooth, but there are some tiny nanoparticles of several nanometers in size located at the surface, which can be assigned as metal Ag nanoparticles. As for TA nanoheterojunctions, TiO_2_ nanoparticles are proportionally located at the surface of Ag_2_V_4_O_11_ nanobelts. Meanwhile, it is worthwhile to note that the amount of TiO_2_ nanoparticles on Ag_2_V_4_O_11_ nanobelts increase with the increase of TiO_2_, and TiO_2_ nanoparticles agglomerate more easily with the increase of TiO_2_. 

**Figure 3:**
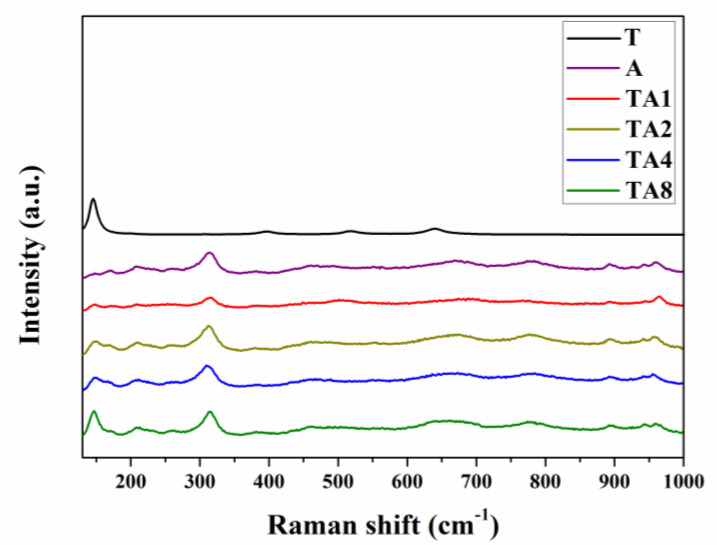
Raman spectra of T, A, TA1, TA2, TA4, and TA8 samples, respectively.

In order to further explore the components of nanoheterojunctions, the Raman and XPS survey spectra are employed. As shown in Figure 3, the Raman shift at 144 cm^−1^ are assigned to the E_g_ mode of anatase TiO_2_; evidently, the peak intensity is enhanced with the increase of TiO_2_ additive amount. Moreover, we employed the EDS test to characterize the elemental composition and real molar ratio of TiO_2_/Ag_2_V_4_O_11_, and the results are presented in Figure S1. It is evident that TiO_2_/Ag_2_V_4_O_11_ is composed of Ti, Ag, V and O elements. According to the EDS results, it can be obtained that the molar ratios of TA1, TA2, TA4, and TA8 are 0.5, 1.5, 3.7 and 6.2, respectively. The reduced amount of TiO_2_ compared with the initial additive amount can be attributed to the loss of uncombinative TiO_2_ nanoparticles.

**Figure 4:**
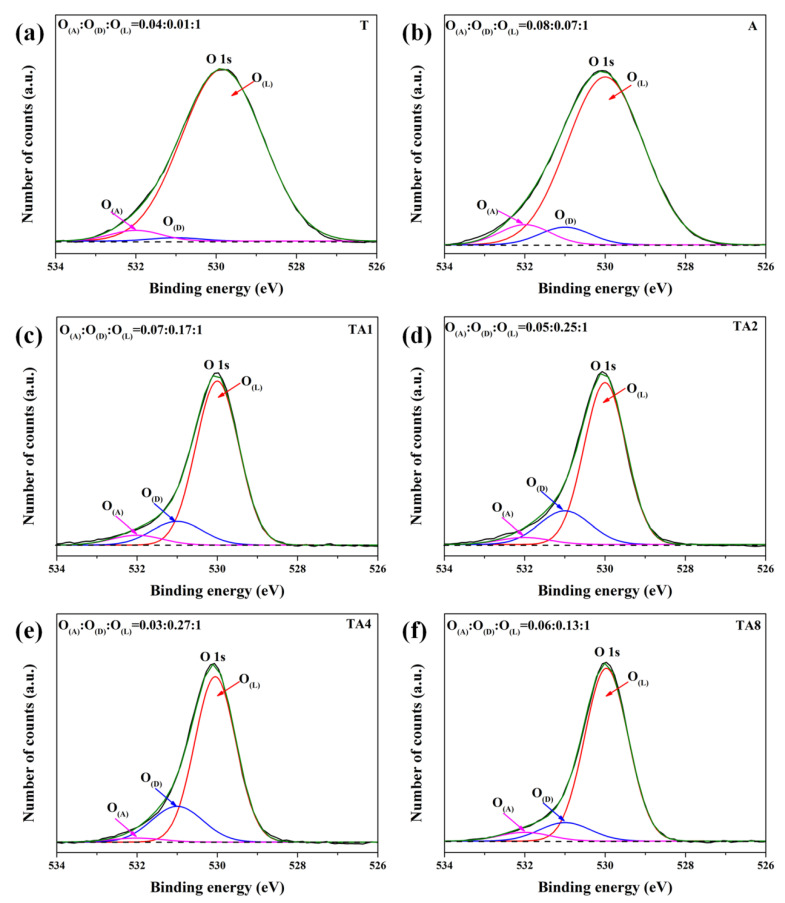
(**a**–**f**) The high-resolution XPS O1s core level spectra of T, A, TA1, TA2, TA4, and TA8 samples, respectively.

In addition, we used XPS characterization to further analyze the elemental composition of TiO_2_/Ag_2_V_4_O_11_. The XPS survey spectra of samples are presented in Figure S2, it is obtained that the nanoheterojunctions are composed of Ti, Ag, V, and O elements, respectively. Furthermore, the high-resolution XPS spectra of samples are employed to analyze the surface composition, as shown in Figure S3. It can be obtained that the Ag, Ag^+^, V^4+^, V^5+^, and Ti^4+^ coexist in the TA samples. Accordingly, the tiny Ag nanoparticles on the surface absorb more visible light owing to the surface plasmon resonance [26], thus the photocatalytic activity is improved. In addition, the existence of V^4+^ indicates the possibility of an oxygen defect, thus we obtained the O 1s core-level spectra to analyze the surface information of oxygen, as shown in Figure 4. The lower binding energy peak located at 530 eV can be assigned to the lattice oxygen (O_(L)_), the peak at 531 eV can be ascribed to the defect oxygen (O_(D)_) of O_2_^2−^/O^−^, and the higher energy peak (532 eV) corresponds to the adsorption oxygen (O_(A)_) and hydroxyl groups (-—OH) on the surface [27]. TA samples possess much more defect oxygen than the pure samples, and the different intensities of the O_(D)_ peak for TA samples should be interrelated with the effective amount of nanoheterojunctions. Hence, we can deduce that the added oxygen defects in the TA samples originate from the interface defects formed at the nanoheterojunctions. The existence of the interface defects exert a peculiar effect on the carrier kinetics, and this will be discussed in detail in the remainder of the paper.

**Figure 5:**
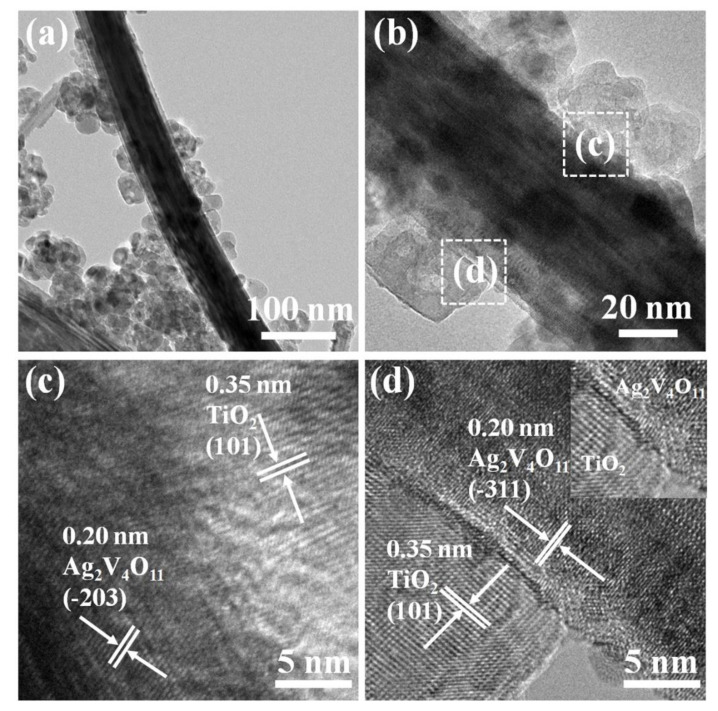
(**a**,**b**) TEM and (**c**,**d**) HRTEM images of TA2 sample.

TEM and HRTEM images were employed to further characterize the morphology and crystal structure of TA nanoheterojunctions. Figure 5a,b show the TEM images of TA2; it can be seen that the Ag_2_V_4_O_11_ nanobelt with a width of about 60 nm is decorated with TiO_2_ nanoparticles. As displayed in Figure 5c,d, the fringe interval of 0.35 nm is assigned to the d-spacing of the (101) crystal plane of anatase TiO_2_, and the lattice distances of 0.31 nm and 0.20 nm correspond to the lattice distances of the (−203) and (−311) planes of monoclinic Ag_2_V_4_O_11_. Furthermore, it is evident that the staggered interface is formed between TiO_2_ and Ag_2_V_4_O_11_ lattices, revealing the tight interfacial contact and the formation of TiO_2_/Ag_2_V_4_O_11_ heterojunction.

**Figure 6:**
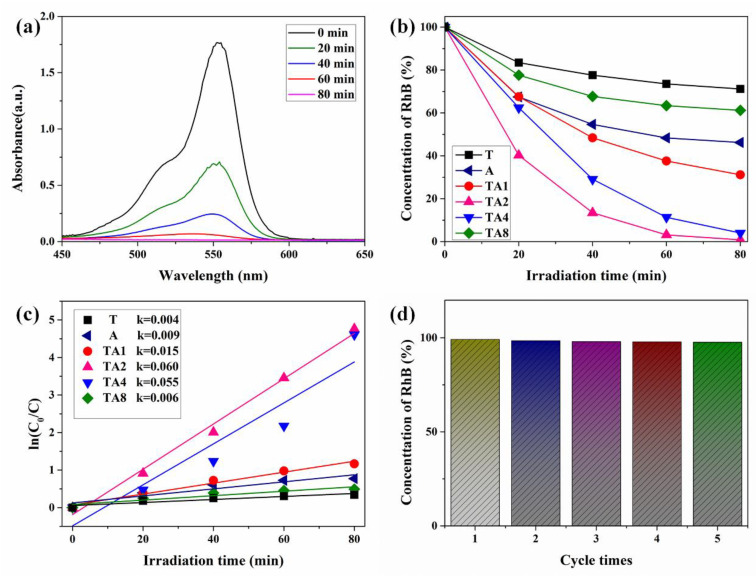
(**a**) Absorption spectral changes of RhB solution with TA2 under visible light irradiation, (**b**) comparison of photocatalytic degradation of RhB and (**c**) the photodegradation rates of samples under visible light irradiation, (**d**) cycling photocatalytic performances of TA2 under visible light irradiation.

In this paper, samples are used to decompose RhB to determine the photocatalytic activity. As shown in Figure 6a, it is obvious that the absorbance of RhB solution in the presence of TA2 decreases with the irradiation time, indicating that the RhB can be effectively decomposed by TA2 under visible irradiation. Furthermore, as shown in Figure 6b, after 80 min irradiation under visible light, the concentration of RhB molecules was decreased to approximately 28.8% by the T sample. This mainly derives from the self-degradation and dye sensitization of RhB [28,29,30,31]. Distinctly, the self-degradation and dye sensitization effect can also exist in other semiconductor catalysts. Furthermore, it can be obtained that the RhB molecules are decomposed to approximately 53.8%, 68.8%, 99.2%, 96.0%, and 38.8% by A, TA1, TA2, TA4, and TA8, respectively. The decomposition rates of T, A, TA1, TA2, TA4, and TA8 are 0.004, 0.009, 0.015, 0.060, 0.055, and 0.006, respectively. Herein, compared with the T sample, TA samples possess much higher photocatalytic activity, indicating that the effect of the heterojunction is much more remarkable than the self-degradation and dye sensitization effect of RhB. Additionally, TA2 shows the highest photocatalytic activity, which can be ascribed to the optimal quantity of nanoheterojunctions. Meanwhile, as shown in Figure 6d, it can be observed that there is only a tiny reduction in the photocatalytic activity after five cycles, indicating that the TA2 exhibits outstanding reusability and stability.

### 3.2. Characterizations of Energy Bands Structure 

The above characterizations reveal that TiO_2_/Ag_2_V_4_O_11_ nanoheterojunctions have been successfully fabricated and possess enhanced visible-light photocatalytic activity. To further understand the mechanism of enhanced photocatalytic performance, we investigated the energy band structure and defect distribution of samples. The standard literature energy levels of TiO_2_ (CB of −4.0 eV, VB of −7.2 eV, and FB of −4.2 eV, vs. the vacuum level, respectively) were employed for the energy band matching analysis [32]. The band gap energy of Ag_2_V_4_O_11_ was obtained from the UV-Vis absorption spectrum according to the Kubelka–Munk (KM) equation:

(1) $$\alpha h\nu =A{\left(h\nu -E_{g}\right)}^{2}$$

**Figure 7:**
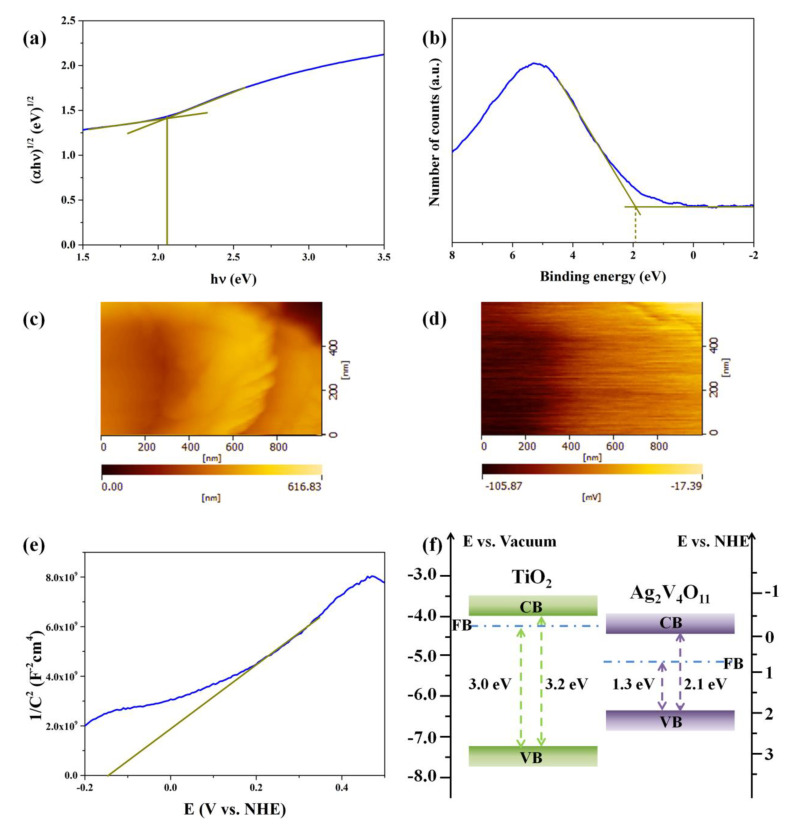
(**a**) The plot of (αhν)^1/2^ vs. photo energy hν of A sample, (**b**) XPS valence band spectrum of A sample, (**c**) AFM images of the A sample deposited on a SiO_2_ substrate, (**d**) Fermi energy level difference between the Rh probe and Ag_2_V_4_O_11_ using KPFM test, (**e**) Mott–Schottky plots of the A sample, (**f**) energy bands of the T and A samples.

**Figure 8:**
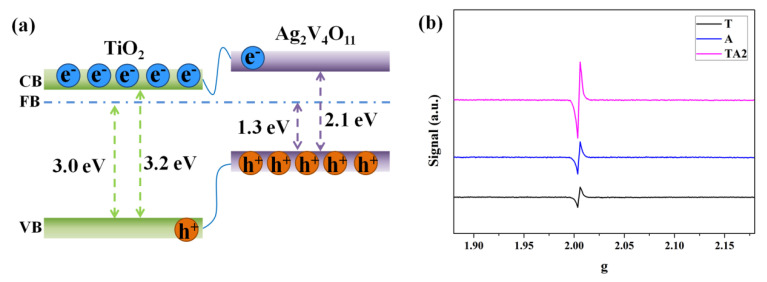
(**a**) Schematic illustration of energy band matching and the proposed mechanism of free electron–hole pairs’ transfer route for TA nanoheterojunctions under visible light irradiation, (**b**) EPR spectra of the T, A, TA2 samples at room temperature.

As shown in Figure 7a, the band gap energy of Ag_2_V_4_O_11_ was estimated to about 2.1 eV from the intercept of the tangent to the plots. Moreover, the valence band edge of Ag_2_V_4_O_11_ was obtained by the XPS valence band spectrum. As shown in Figure 7b, the valence band of Ag_2_V_4_O_11_ is located at 1.9 eV vs. NHE, equally, the VB of Ag_2_V_4_O_11_ is approximately −6.4 eV vs. vacuum. Furthermore, the Fermi energy test was performed on the Ag_2_V_4_O_11_ film sample using a KPFM test. Figure 7c displays the corresponding atomic force microscopy (AFM) scanning image of the Ag_2_V_4_O_11_ film, the film is rough, and the thickness is approximately 300–600 nm. Figure 7d shows the Fermi energy level difference between the Rh probe and the Ag_2_V_4_O_11_ film, it is evident that the Fermi energy band of Ag_2_V_4_O_11_ sample is approximately 0.05–0.1 eV lower than the Rh probe; the undulation of the Fermi energy level difference can be attributed to the variation in the film’s roughness. Considering that the Femi energy value of Rh is −5.0 eV vs. vacuum, the Femi energy of Ag_2_V_4_O_11_ sample is about −5.1 eV vs. vacuum. In addition, the Mott–Schottky plot of Ag_2_V_4_O_11_ is displayed in Figure 7e; it can be obtained that the flat band potential of Ag_2_V_4_O_11_ sample is approximately −0.15 eV vs. NHE. Herein, according to the above comprehensive analysis, the energy bands of Ag_2_V_4_O_11_ samples (CB of −4.3 eV, VB of −6.4 eV, and FB of −5.1 eV, vs. the vacuum level, respectively) are obtained and displayed in Figure 7f. When forming the TA nanoheterojunctions, the Fermi energy bands of TiO_2_ and Ag_2_V_4_O_11_ should be aligned at the same level; therefore, both the conduction band and valence band of Ag_2_V_4_O_11_ are located above those of TiO_2_, as presented in Figure 8a. When the TA nanoheterojunctions were excited by visible light, many photogenerated electrons are excited to the conduction band of Ag_2_V_4_O_11_. At this point, the excited free electrons then quickly transfer to the conduction band of TiO_2_ because of the nanoheterojunction. According to the previous reports, the transfer time for this process often takes place at the ps scale [33]; therefore, the nanoheterojunctions possess a longer recombination time compared to the bare samples. Furthermore, EPR spectra are employed to investigate the vacancy defects of samples (Figure 8b), where the peak located at g = 2.001 can be attributed to the oxygen vacancies [34]. It is evident that the TA2 sample exhibits a greatly enhanced intensity of the profiles, demonstrating that abundant oxygen vacancies are formed at the interfaces of the nanoheterojunctions, and the result is consistent with the XPS results. Therefore, it can be obtained that photogenerated carriers can be effectively separated because of the nanoheterojunction effect, while the increased defects at the interface would capture the electrons. Herein, it must be further investigated what effects the nanoheterojunction effect and interface defects have on the carriers’ kinetics.

### 3.3. Characterization of the Carrier Kinetics

**Figure 9:**
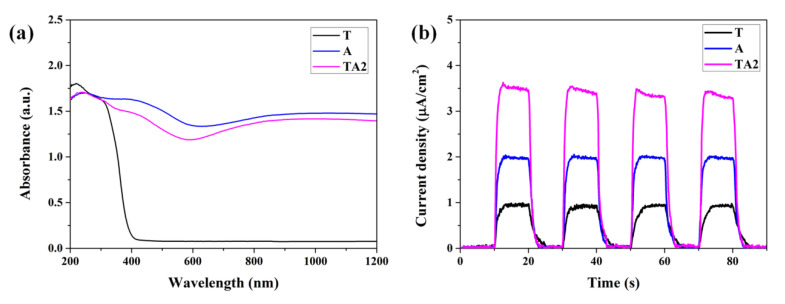
(**a**) UV-Vis absorbance spectra and (**b**) photocurrent responses of the T, A, and TA2 samples.

Based on the above results and analysis, we further investigate the photogenerated charge kinetics of electron–hole pairs in the generation, transportation, and recombination processes. Firstly, the absorption ability of samples is an important factor to evaluate the photocatalytic activity, as displayed in Figure 9a, it can be observed that T sample exhibits a sharp absorption edge at approximately 400 nm because of the large band gap of TiO_2_. As for the A and TA2 samples, the absorption abilities to visible light are effectively improved compared to pure TiO_2_, which are benefited by the narrow band gap of Ag_2_V_4_O_11_. Additionally, we employed the photoelectrochemical measurements to obtain the transient photocurrent responses of the samples. As shown in Figure 9b, TA2 nanoheterojunctions show a much higher photocurrent intensity and faster photocurrent response than that of the T and A samples, indicating that TA2 nanoheterojuntions exhibit higher photocarrier generation and quicker collection dynamics. 

**Figure 10:**
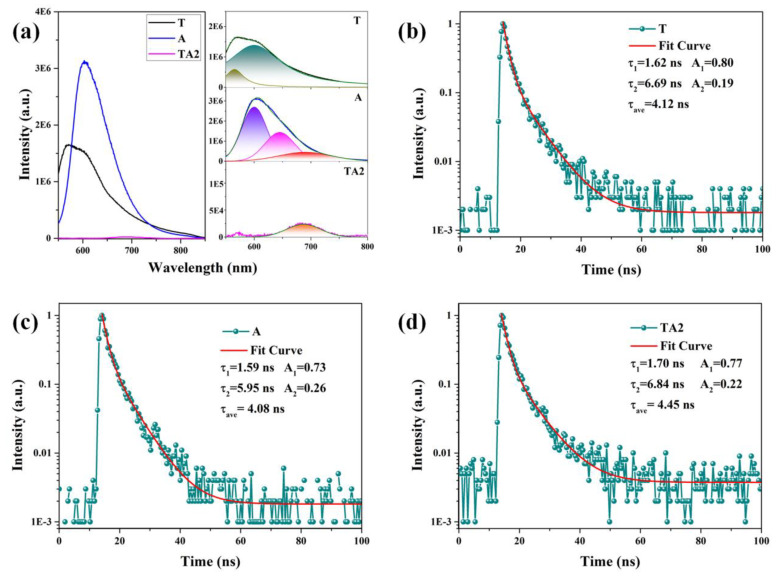
(**a**) PL spectra and fitted peaks of the T, A and TA2 samples, (**b**–**d**) TRPL spectra of the T, A, and TA2 samples, respectively.

In order to track the kinetics of photogenerated electron–hole pairs, PL spectra tested under a 532 nm laser excitation are applied for analysis, as shown in Figure 10a. TA2 nanoheterojunctions show apparently lower PL intensity than that of T and A samples, indicating that the recombination of photogenerated electron–hole pairs in TA2 nanoheterojunctions can be effectively inhibited. Moreover, the PL peaks of the T and A samples were concretely fitted for deeper analysis. The fitted PL peaks of the T sample located at 565 nm (2.19 eV) and 600 nm (2.07 eV) correspond to the SRH recombination between electrons at defect levels and holes at valence level. As for the A sample, the PL peak at 600 nm (2.07 eV) can be assigned to the band gap recombination of the photogenerated carrier, and the PL peaks located at 645 nm (1.92 eV) and 690 nm (1.88 eV) originate from the SRH recombination. When it comes to TA nanoheterojunctions, the strong emissions of T and A are quenched; only a low-intensity emission peak located at 687 nm can be observed, revealing that the recombinationroutes in T or A are prevented in the TA2 nanoheterojunctions.

Additionally, we investigate the TRPL spectra of samples at 600 nm excited by a 532 nm laser to further understand the recombination kinetics of photogenerated carriers. The decay of the photoluminescence intensity with time indicates the quenching of photogenerated carriers owing to recombination, and the TRPL decay profiles are fitted using a double-exponential function as follows [35]:

I_(t)_ = A_1_exp(−t/τ_1_) + A_2_(−t/τ_2_) (2)

where I_(t)_ is intensity, A_1_ and A_2_ are relative magnitudes of τ_1_ and τ_2_, and τ_1_ and τ_2_ are the photoluminescence decay time. As shown in Figure 10b–d, the values of τ_1_ for T, A, and TA2 are 1.62 ns, 1.59 ns, and 1.70 ns, respectively, which correspond to the photoluminescence of free exciton states. The values of τ_2_ for T, A, and TA2 are 6.69 ns, 5.95 ns, and 6.84 ns, respectively, and the relatively slow time scale of photoluminescence can be explained by the later SRH recombination of holes and trapped electrons. Moreover, it can be obtained that the TA2 nanoheterojunctions display prolonged emission lifetimes compared to that of T and A samples, and recombination of free charges plays a major role in the resulting photoluminescence in the T, A, and TA2 samples.

### 3.4. Discussions

**Figure 11:**
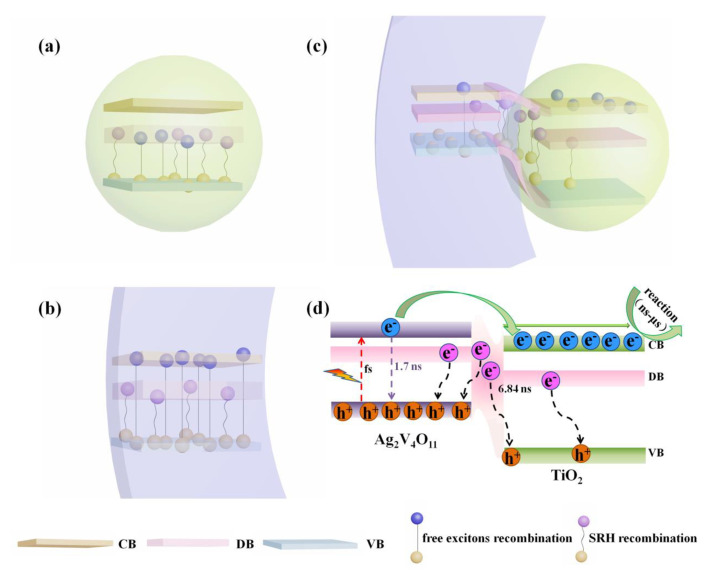
Schematic illustration of the carrier recombination kinetics occurred in the conduction band (CB), defect band (DB), and valence band (VB) of the (**a**) T, (**b**) A, and (**c**,**d**) TA samples, respectively.

Significantly, TA nanoheterojunctions manifest much higher photocarrier generation and faster collection kinetics owing to the nanoheterojunction effect. This is because that both the nanoheterojunction effect and interface defects have effects on recombination kinetics. Thus, we further systematically discussed the carrier kinetics, and the schematic illustration of the T, A, and TA nanoheterojunctions are presented in Figure 11. As shown in Figure 11a, in virtue of the large bandgap energy of TiO_2_, the electrons in the VB can are not easily excited to the CB by visible light’ only a minority of the electrons can be excited to the defect band (DB). Considering the poor photocatalytic activity and strong defect luminescence intensity of TiO_2_ under visible light irradiation, we can deduce that most of those electrons in the DB would be quenched by free exciton recombination and SRH recombination. Apropos of Ag_2_V_4_O_11_, the electron–hole pairs were generated very rapidly under visible light irradiation. Then, the carriers were expended through three modes: some of the electrons transfer to the surface for chemical reactions as embodied in photocatalytic activity; some free electrons can recombine with holes quickly through direct transition, corresponding to the band gap luminescence peak in PL spectra; another portion of the electrons were captured by the oxygen vacancy defects during the diffusion process and ultimately, participated in photocatalytic reactions or underwent SRH recombination, as shown in Figure 11b. As for TA nanoheterojunctions, photogenerated electrons in CB of Ag_2_V_4_O_11_ can quickly transfer to the CB of TiO_2_ owing to the nanoheterojunction effect; the transfer process usually occurs within dozens of picoseconds [34,36]. Notably, the fast carrier separation and quick transfer prolonged the time scale of free exciton recombination. In addition, as revealed by EPR spectra and the prolonged SRH recombination time, many oxygen defects exist in the nanoheterojunction interface of the TA samples; these additional defects captured some of the electrons in their transfer process, thus resulting in additional SRH recombination, as shown in Figure 11c,d. Considering the prolonged overall SRH recombination time of the TA sample (6.84 ns), we can deduce that the SRH recombination stemming from interface defects is much more long-term compared to that from the two bare samples. Accordingly, thanks to the effective carriers’ separation and additional electron capture, the TA nanoheterojunctions show a prolonged carrier lifetime compared to pure components, and provide enough time for electron transfer and photocatalytic reaction, leading to an enhancement on the photocatalytic activity. Herein, to sum up, the enhanced photocatalytic mechanisms of TiO_2_/Ag_2_V_4_O_11_ can be attributed to the broadening of the absorption region, higher photocarrier generation, effective carrier separation, longer carrier lifetime, and faster collection dynamics.

## 4. Conclusions

In summary, TiO_2_/Ag_2_V_4_O_11_ nanoheterojunctions with enhanced visible light photocatalytic activity have been successfully synthesized, in which TA2 shows the highest photocatalytic activity. Furthermore, the carrier recombination dynamics of samples are revealed by PL and TRPL spectra, which were shown to be improved by the construction of the nanoheterojunctions; the free exciton recombination of the TA2 sample occurs in approximately 1.7 ns, which is longer than that of bare samples, and the time scale for Shockley–Read–Hall of photogenerated electrons and holes is prolonged to 6.84 ns, indicating that TA2 possesses effective electron transfer and capture abilities, thus the electrons have a sufficient lifetime to participate in the photocatalytic reactions. In addition, besides the excellent carrier transfer and capture dynamics, the enhanced photocatalytic mechanisms of TiO_2_/Ag_2_V_4_O_11_ can also be attributed to the synergistic effect of the broadened absorption region, higher photocarrier generation, and faster collection dynamics. This research reveals that both the energy matching and interface defects have effects on the recombination kinetics of nanoheterojunctions, providing a further understanding of the carrier kinetics of nanoheterojunctions and providing significant guidance for the construction of nanoheterojunctions with higher photocatalytic activity.

## Supplementary Materials

The following are available online at https://www.mdpi.com/2079-4991/10/5/828/s1, Figure S1 (a-d) EDS spectra of TA1, TA2, TA4, and TA8 samples, respectively. Figure S2 (a-f) XPS survey spectra of T, A, TA1, TA2, TA4, and TA8 samples, respectively. Figure S3 The high resolution XPS core-level binding energy spectra of samples. (a) Ti 2p spectra of T sample; (b-c) Ag 3d and V 2p spectra of A sample; (d-o)Ti 2p, Ag 3d, V 2p spectra of (d-f) TA1, (g-i) TA2, (j-l) TA4, and (m-o) TA8 samples, respectively.
