# Supplementary material for: Carrier Transfer and Capture Kinetics of the TiO2/Ag2V4O11 Photocatalyst
Source: Nanomaterials (Basel). 2020 Apr 27;10(5):828. doi: 10.3390/nano10050828 (PMC7711476; doi:10.3390/nano10050828)
Supplement: Supplementary file 1 [file nanomaterials-10-00828-s001.pdf]

Supplementary Materials:

Article

Carrier Transfer and Capture Kinetics of the $TiO_2/Ag_2V_4O_{11}$ Photocatalyst

Yun Zhou [1,2,†], Qiujie Ding [1,2,†], Yuan Wang [2,*], Xiaoping OuYang [1,*], Lixin Liu [2], Junyu Li [1,2] and Bing Wang [1,2]

[1] School of Materials Science and Engineering, Xiangtan University, Xiangtan 411105, Hunan, China zhouyun720@126.com (Y.Z.); xtdxdqj@sina.com (Q.D.); li_junyu@nuaa.edu.cn (J.L.); wangbing2015@aliyun.com (B.W.)

[2] Institute of Fluid Physics, China Academy of Engineering Physics, P.O. Box 919-111, Mianyang, Sichuan 621900, People's Republic of China; liulix00@gmail.com

* Correspondence: wangyuan0000@gmail.com (Y.W.); oyxp2003@aliyun.com (X.O.;) Tel.: +86 0816 248139

† These authors contributed equally to this work.

The XPS survey spectra of samples are presented in Figure S1. As shown in Figure S1a, the T sample is composed of Ti and O elements. Ag, V, and O elements appear in the A sample, as displayed in Figure S1b. Moreover, as shown in Figure S1c-f, it is obtained that the TA samples are composed of Ti, Ag, V, and O elements, respectively. The intensity of the Ti 2p peaks were enhanced with the increase of TiO_2 additive amount. The atomic percentages were obtained from XPS results; it was calculated that the real $TiO_2/Ag_2V_4O_{11}$ molar ratio of TA1, TA2, TA4, and TA8 were about 3.85, 4.55, 5.92, and 12.07, respectively. The higher molar ratio values compared to the added quantity is attributed to the reaction loss of $Ag_2V_4O_{11}$ during the hydrothermal synthesis. Additionally, compared with the V atomic percentage, the surplus of Ag atom also reflects the existence of metal Ag.

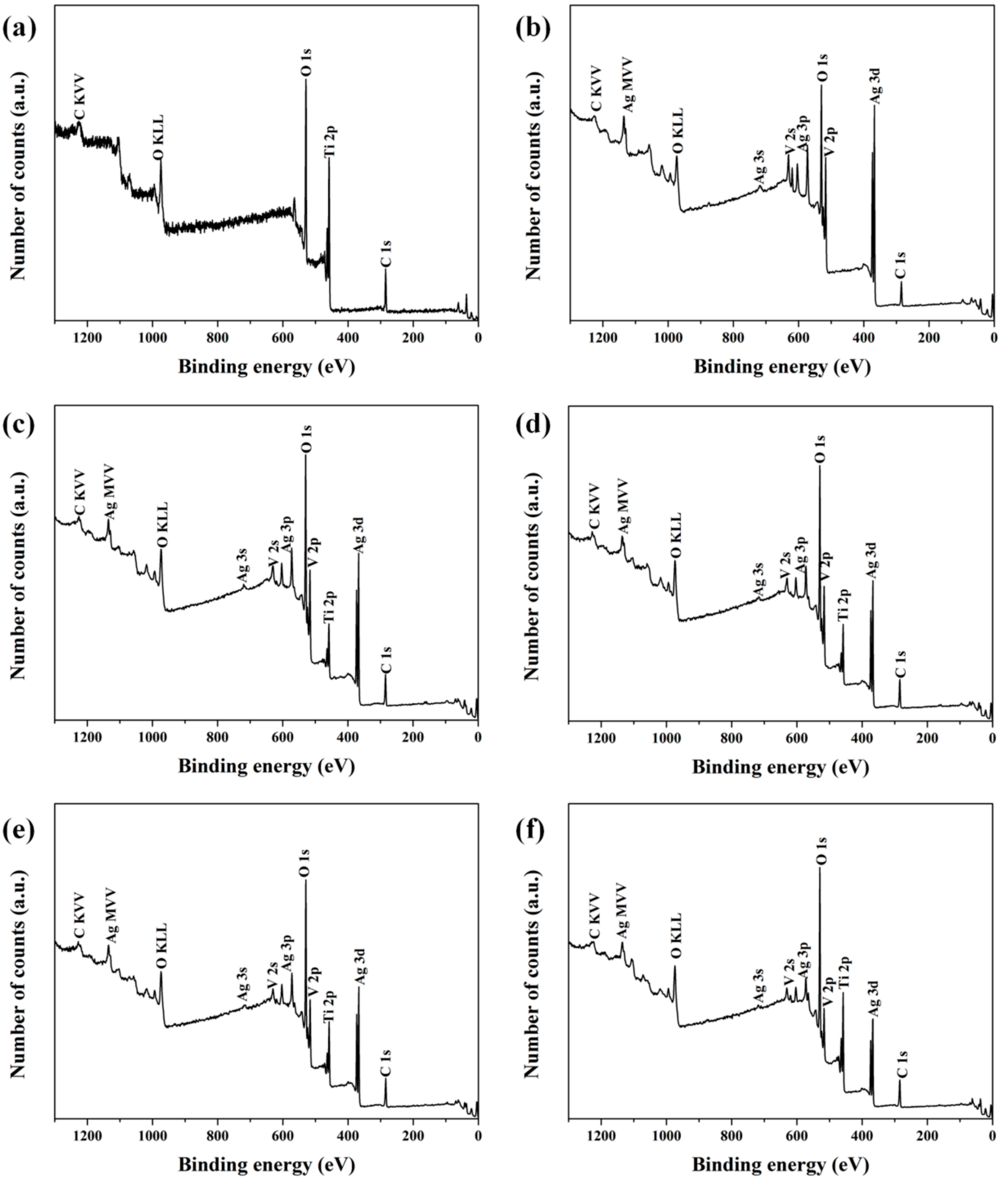

Figure S1 (a-d) XPS survey spectra of T, A, TA1, TA2, TA4, and TA8 samples, respectively.

Figure S2a shows the high-resolution XPS spectra of Ti 2p core-level binding energy of T samples. The peaks located at 464.2 and 458.6 eV can be ascribed to the Ti 2p1/2 and Ti 2p3/2 spin–orbital components of Ti^{4+} [1]. Figure S2b-c shows the high-resolution XPS spectra of Ag 3d and V 2p core-level binding energy of the A samples. As shown in Figure S2b, the peaks located at 367.8 eV and 373.8 are characteristic of Ag^{+}, and the peaks at 368.4 eV and 374.4 eV can be ascribed to metallic Ag [2]. The V 2p core-level spectrum of the A sample is shown in Figure S2c,

the peaks at 524.5 eV and 517 eVare characteristic of a +5 oxidation state of vanadium, and the two low-intensity peaks located at 523 eV and 515.5 eV are ascribed to V^{4+} [3]. It can be obtained that the Ag, Ag^{+}, V^{4+}, and V^{5+} coexist in the TA samples. Figure S2d-o shows the high-resolution XPS spectra of Ti 2p, Ag 3d, V 2p, and O 1s core-level binding energy of TA1,TA2, TA4, and TA8, respectively. It is evident that the Ag, Ag^{+}, V^{4+}, V^{5+}, and Ti^{4+} coexist in the TA samples.

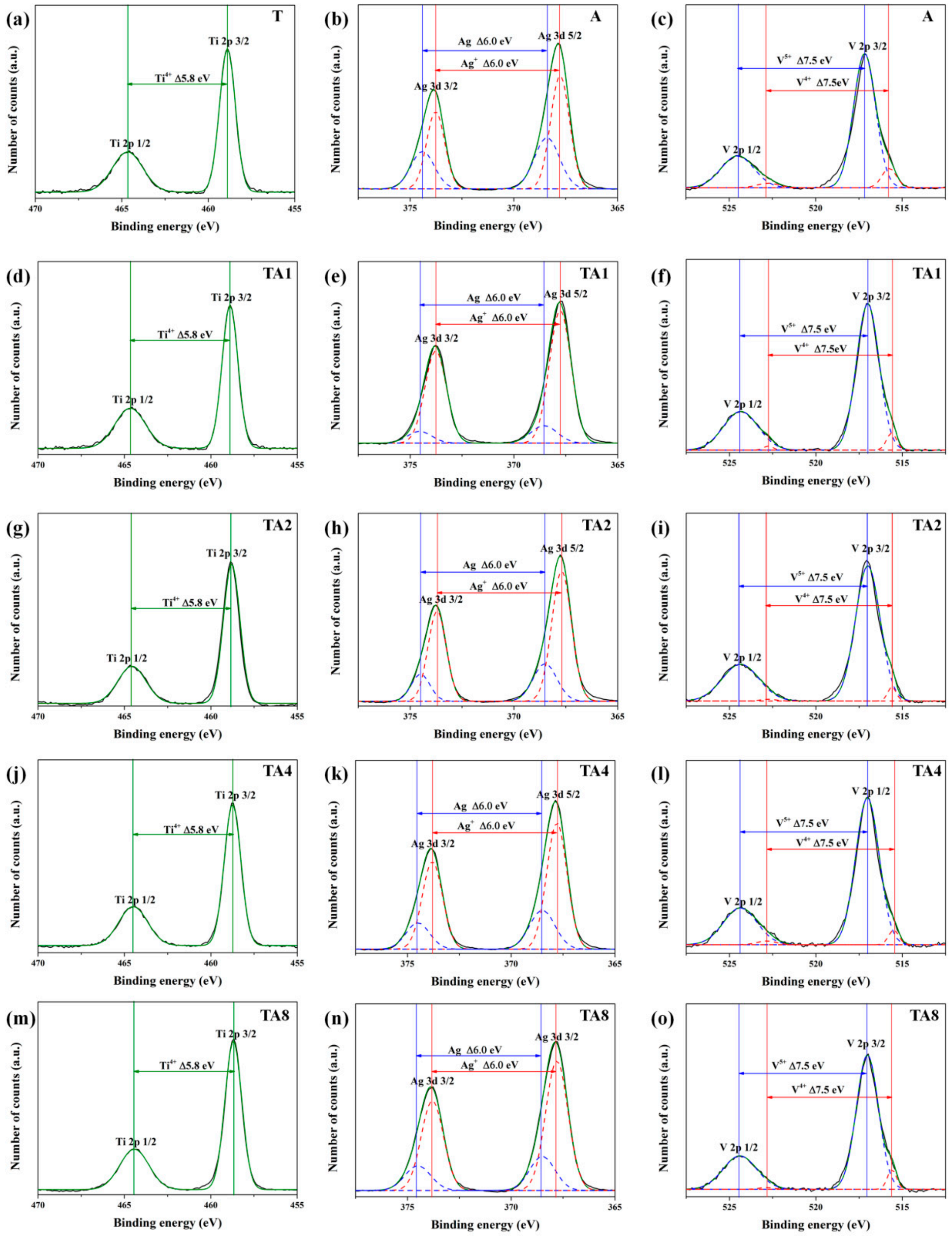

Figure S2 The high-resolution XPS core-level binding energy spectra of samples. (a) Ti 2p spectra

of T sample; (b-c) Ag 3d and V 2p spectra of the A sample; (d-o)Ti 2p, Ag 3d, V 2p spectra of the (d-f) TA1, (g-i) TA2, (j-l) TA4, and (m-o) TA8 samples, respectively.

References:

[1] Chen, X.; Liu, L.; Peter, Y.Y.; Mao, S.S. Increasing solar absorption for photocatalysis with black hydrogenated titanium dioxide nanocrystals. *Science*, **2011**, *331*, 746–750.

[2] Rocha, T.C.R.; Oestereich, A.; Demidov, D.V.; Hävecker, M.; Zafeiratos, S.; Weinberg, G.; Bukhtiyarov, V.I.; Knop-Gericke, A.; Schlögl, R. The silver–oxygen system in catalysis: new insights by near ambient pressure X-ray photoelectron spectroscopy. *Phys. Chem. Chem. Phys.* **2012**, *14*, 4554-4564.

[3] Wang, Y.; Liu, L.; Meng, C.; Zhou, Y.; Gao, Z.; Li, X.; Cao, X.; Xu, L.; Zhu, W. A novel ethanol gas sensor based on $TiO_2/Ag_{0.35}V_2O_5$ branched nanoheterostructures. *Sci. Rep.* **2016**, *6*, 33092.
